# Impairment of Vowel Articulation as a Possible Marker of Disease Progression in Parkinson's Disease

**DOI:** 10.1371/journal.pone.0032132

**Published:** 2012-02-28

**Authors:** Sabine Skodda, Wenke Grönheit, Uwe Schlegel

**Affiliations:** Department of Neurology, Knappschaftskrankenhaus, Ruhr-University of Bochum, Bochum, Germany; University of Leicester, United Kingdom

## Abstract

**Purpose:**

The aim of the current study was to survey if vowel articulation in speakers with Parkinson's disease (PD) shows specific changes in the course of the disease.

**Method:**

67 patients with PD (42 male) and 40 healthy speakers (20 male) were tested and retested after an average time interval of 34 months. Participants had to read a given text as source for subsequent calculation of the triangular vowel space area (tVSA) and vowel articulation index (VAI). Measurement of tVSA and VAI were based upon analysis of the first and second formant of the vowels /α/, /i/and /u/ extracted from defined words within the text.

**Results:**

At first visit, VAI values were reduced in male and female PD patients as compared to the control group, and showed a further decrease at the second visit. Only in female Parkinsonian speakers, VAI was correlated to overall speech impairment based upon perceptual impression. VAI and tVSA were correlated to gait impairment, but no correlations were seen between VAI and global motor impairment or overall disease duration. tVSA showed a similar reduction in the PD as compared to the control group and was also found to further decline between first and second examination in female, but not in male speakers with PD.

**Conclusions:**

Measurement of VAI seems to be superior to tVSA in the description of impaired vowel articulation and its further decline in the course of the disease in PD. Since impairment of vowel articulation was found to be independent from global motor function but correlated to gait dysfunction, measurement of vowel articulation might have a potential to serve as a marker of axial disease progression.

## Introduction

Hypokinetic dysarthria in Parkinson's disease (PD) is a multidimensional impairment affecting all different aspects of speech as speech respiration, phonation, articulation and prosody [1], [2]. Imprecise vowel articulation has been shown to be present even in mild stages of PD [3] and commonly contributes to reduced speech intelligibility [1], [4], [5]. Kinematic and acoustic measurements revealed that PD patients produce “undershooting” of articulatory gestures [1], [6], [7] which lead amongst others to imprecise articulation of consonants and vowels [8], [9]. Furthermore, several studies provide kinematic evidence of reduced amplitude and velocity of lip, tongue and jaw movements (the so called “articulators”), which may represent the physiological basis of hypokinesia and rigidity of the vocal tractus [6], [10], [11] or may be related to deficits in scaling amplitude, impaired internal cueing and abnormal perception [12]. Evidence from acoustic studies also supports the conclusion that the reduced range of articulator movements in PD leads to imprecise vowel articulation caused by impaired and less distinctive “formant” generation [13].

**Figure 1:**
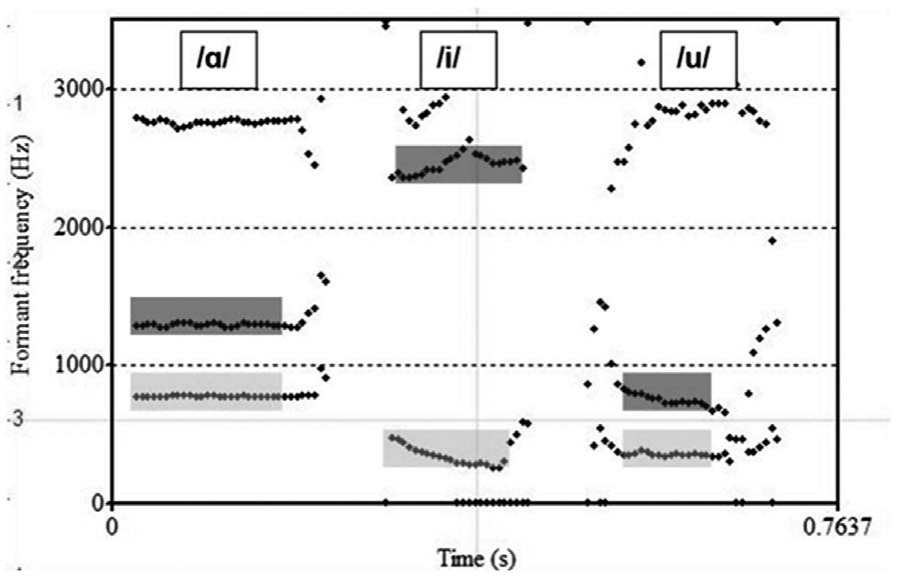
Exemplified comparison of the first and second formant frequencies (F1 and F2) of the vowels /a/, /i/ and /u/ of a healthy speaker. The F1 and F2 frequencies are marked in light (F1) and dark (F2) grey. x-axis: time; y-axis: frequency (in hertz).

**Figure 2:**
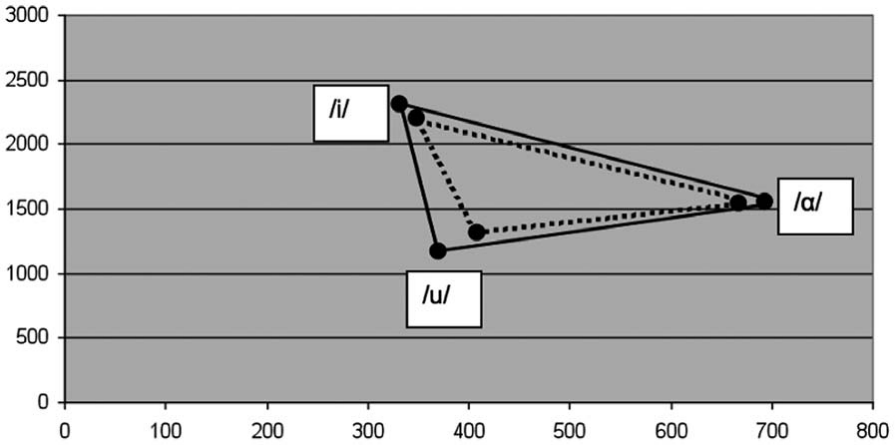
Exemplified triangular vowel space area (tVSA) of a healthy speaker and a patient with PD (dotted triangle).

Vowels are formed primarily by movements of the articulators creating oropharyngeal resonating cavities which amplify certain frequency bands of the voice spectrum. These harmonics (the so called “formants”) define the single vowels by their typical distinct peaks of acoustic energy. The position of the articulators therefore defines the three dimensional characteristics of the vocal tractus and influences the formant frequencies, especially of the first (F1) and second (F2) formant. Frequencies of F1 and F2 are mainly defined by the tongue position with the simplified “rule” that the F1 frequency is inversely related to the height of the tongue whereas the F2 frequency is directly related to the frontness of the tongue position [14]. As a consequence, limited movements of the articulators and particularly of the tongue, as suggested in PD, lead to inadequate vowel formation by a restriction of formant production which should be characterized by a lowering of normally high frequency formants and by an elevation of normally low frequency formants [15]. This hypothesised constriction of working space for vowels in PD should be mirrored by a reduction of the triangular vowel space area (tVSA) which can be assessed by plotting the F1 frequency as a function of F2 frequency for the three corner vowels /α/, /i/ and /u/ to provide a graphic display of a vowel triangle (see figures 1 and 2). The area of the vowel triangle can be calculated according to the following formula: tVSA = abs((F1_/i/ * (F2_/α/−F2_/u/)+F1_/α/ * (F2_/u/−F2_/i/)+F1_/u/ * (F2_/i/−F2_/α/)/2). The absolute Hz^2^ values of vowel area obtained through this calculation do not possess functional significance on their own, although they are estimated to serve as an index of the general pattern of change in the working space for vowels [16], [17], [18]. However, measurement of the triangular or quadrilateral VSA, although well established as the most common acoustic metric in research on disturbed vowel articulation (e.g. [13], [19]), has been proven to be insensitive to mild or moderate forms of dysarthria, especially in PD patients [4]. Moreover, in some studies, the VSA accounted for only about 10% of the variance in measures of speech intelligibility [20], [21]. Recently, a further surrogate parameter called “vowel articulation index/VAI” (and its reciprocal value, the so called “formant centralization ratio/FCR”) had been developed by Sapir and coworkers [15], [18] and been proven by empirical testing to be more sensitive in Parkinsonian hypokinetic dysarthria than tVSA in several publications [15], [18], [22]. VAI can be calculated by the following formula: VAI = (F2/i/+F1/α/)/(F1/i/+F1/u/+F2/u/+F2/α/). Formant concentration caused by a reduction of articulator movements in Parkinsonian speakers is therefore expected to lead to a decrease of the numerator (F2/i/+F1/α/) and an increase of the denominator (F1/i/+F1/u/+F2/u/+F2/α/) resulting in an overall reduction of VAI. This hypothesis has been confirmed in a previous investigation of VAI in Parkinsonian patients without any or only mild degrees of dysarthria [3].

According to a previous investigation on a large sample of speakers with PD, disturbance of voice may be an early and predominant feature and is complemented by additional impairment of fluency and articulation in the more severe stages of the disease [23]. Concerning overall motor deterioration in PD, global motor function was reported to show an annual decline of about 3% in one population-based study [24]; though, different courses of disease progression were found when related to the age of onset with a faster decline of mentation and gait in the older-onset group [25]. On the other hand, positron emission tomographic imaging/PET-based studies suggested a negative exponential course of progression at least when related to dopaminergic neurodegeneration [26], [27]. These ostensible discrepancies between the clinical course of disease progression and findings of functional imaging based investigations might be explained by the fact that PET studies are restricted to the monitoring of defined regions of interest and neurotransmitter systems which do not necessarily mirror the overall disease progression observed in clinical surveys. Besides these considerations about overall disease progression in PD, little is known about the development of different speech modalities in the course of the disease in the individual patient with only single studies documenting a deterioration of distinct prosodic speech dimensions as pitch variability, speech rate and stability of syllable repetition which seem to rather arise after a longer disease duration without correlation to global motor function [28], [29].

To gain additional insight into the development of further aspects of speech in PD, the aim of the present study was the investigation of vowel articulation in the course of the disease in the individual patient and to test for correlations with global motor, gait and speech impairment. According to our hypothesis, a deterioration of global speech impairment in the course of the disease as assessed by perceptual rating should be mirrored by a decrease of tVSA and VAI as surrogate parameters for distinctiveness of vowel articulation. A second aim of the present investigation was to survey, if measurement of VAI turns out to be superior to tVSA in the detection of subtle changes of vowel articulation over time as it has to be supposed according to previous studies.

## Methods

Our study was in compliance with the Helsinki Declaration and had been approved by the Ethics Committee of the Ruhr University Bochum. Written informed consent was obtained from each participant.

From 2002 to 2011, 67 patients (42 male, 25 female) with *idiopathic Parkinson's disease (PD)* were recruited for this study. The diagnosis of PD was based upon clinical criteria according to the UK Parkinson's Disease Society Brain Bank Criteria [30]. Patients' age on first examination ranged from 40 to 80 years (mean: 66.27/median: 67/SD: 7.95). Idiopathic Parkinson's disease had been diagnosed from 1 to 20 years prior to the first examination (mean 6.12/median 5/SD: 4.53). Time between first and second examination ranged from 12 to 88 months (mean: 34.06/median: 30/SD: 20.35). On both visits, each patient underwent a neurological examination, according to UPDRS Motor Score/UPDRS III (UPDRS_t0_: mean: 19.48/median: 18/SD: 10.74/range: 4 to 57 points; UPDRS_t1_: mean: 19.10/median: 18/SD: 8.28/range: 5 to 40 points) and was scored according to Hoehn&Yahr Scale (H&Y_t0_: mean: 2.07/median: 2/SD: 0.36/range: 1.5 to 3 points; H&Y_t1_: mean: 2.57/median: 2.5/SD: 0.63/range: 1.5 to 4 points) before performing the speech task. Item 18 of the UPDRS Motor Score (“speech”) was taken for global perceptual description of patients' speech (UPDRS_speech_t0_: mean: 0.96/median: 1/SD: 0.66/range: 0 to 2 points; UPDRS_speech_t1_: mean: 1.36/median: 1/SD: 0.87/range: 0 to 3 points). Items 27–30 were taken for the description of posture and gait (UPDRS_gait_t0_: mean: 1.52/median: 1/SD: 1.41/range: 0 to 5 points; UPDRS_gait_1_: mean: 2.78/median: 2/SD: 1.68/range: 0 to 7 points). Furthermore, levodopa equivalence doses/LED were given for the first and second examination [31] (LED_t0_: mean: 563 mg/median: 550 mg/SD: 166/range: 175 to 900 mg; LED_t1_: mean: 723 mg/median: 700 mg/SD: 207; range: 300 to 1325 mg).

A subgroup of 38 patients and 15 controls had participated in a previous study on speech performance [28]. At the time of the examination, patients were on stable dopaminergic medication since at least 4 weeks prior to the examination. Speech and motor examinations were performed 60 to 90 minutes after the morning dose of medication to ensure the “on”-state. None of the patients experienced orofacial or abdominothoracic peak-dose dyskinesia during the examination. Medication with anticholinergics, cholinesterase inhibitors and atypical neuroleptics and severe dementia (MMSE<25 pts.) were exclusion criteria.

As *control group* we tested and re-tested 40 age-matched healthy persons (mean age 67.69 years/median 67.5 years/SD: 6.10/range 55 to 80 years; 20 male, 20 female) which were re-tested after a mean time period of 21.36 months (median 20/SD 6.16/range 12 to 36 months).

None of the participants suffered from relevant hearing impairment as assessed by a hearing screening test (exposition to test sounds prior to the definite examination).

Each participant had to perform a standardized reading task composed of four complex sentences which had been used by our group in previous research on Parkinsonian dysarthria [3], (Supporting Information S1). In order to get more familiar with the text and to exclude difficulties in reading, the participants had to read the text twice; the second sequence was taken for the definite analysis. All participants were native German speakers.

Speech samples were digitally recorded and anonymized by our Parkinson nurse in a quiet room using a commercial audio software (Steinberg WaveLab©, Steinberg Media Technologies GmbH, Hamburg, Germany) and a head-set microphone (Plantronics Audio 550 DSP©, Plantronics Inc., California 95060, USA) positioned 5 cm from the lips. The data were digitized at a sampling rate of 44.1 kHz. Each of the vowels /α/, /i/ and /u/ were extracted 10 times from different defined words within the text. The formant frequency values F1 and F2 were measured separately for each vowel for a 30 ms segment at the temporal midpoint using a special speech software (Praat©) [32]. For each vowel, the average formant frequency values of F1 and F2 were calculated based upon the separate ten measurements (tables 1+2). These average values were taken for the calculation of tVSA and VAI. The examiner who performed the acoustical analysis (S.S.) was blind to participants' condition.

**Table 1:** Male participants: average formant frequency values which were the source for the calculation of VAI and tVSA (table 3).

	PD male (n = 42)				Control male (n = 20)				published data [27]	
	t0		t1		t0		t1			
	mean	S.D.	mean	S.D.	mean	S.D.	mean	S.D.	mean	S.D.
F1_/α/	524.45	86.13	559.24	92.35	582.58	94.69	577.43	96.14	694	95
F2_/α/	1330.86	108.59	1400.93	131.93	1322.49	109.85	1330.01	99.72	1372	153
F1_/i/	312.47	51.21	386.99	223.03	305.31	35.82	311.52	42.11	369	111
F2_/i/	1979.96	96.57	1997.16	139.04	2023.17	109.68	2009.22	101.34	1902	207
F1_/u/	378.90	92.26	456.87	135.00	389.78	73.60	382.65	76.44	310	82
F2_/u/	1320.65	220.06	1573.35	279.30	1376.20	280.63	1385.64	261.62	854	205
								**VAI** 0.894		
								**tVSA** 185,935		

**Table 2:** Female participants: average formant frequency values which were the source for the calculation of VAI and tVSA (table 4).

	PD female (n = 25)				Control female (n = 20)				published data[27]	
	t0		t1		t0		t1			
	mean	S.D.	mean	S.D.	mean	S.D.	mean	S.D.	mean	S.D.
F1_/α/	671.89	75.93	665.95	76.11	701.89	85.19	692.71	81.81	836	135
F2_/α/	1552.12	80.79	1552.81	77.64	1550.07	92.29	1561.22	89.32	1586	156
F1_/i/	334.63	28.91	347.56	30.37	328.11	45.57	331.12	50.10	433	85
F2_/i/	2221.39	174.18	2204.85	163.40	2323.01	118.27	2319.29	111.74	2095	259
F1_/u/	359.01	46.94	408.09	57.09	371.70	69.72	369.42	61.16	442	85
F2_/u/	1169.39	120.49	1315.87	117.78	1171.02	141.45	1180.82	132.11	1081	183
								**VAI** 0.827		
								**tVSA** 100,273		

Winstat© (Bad Krotzingen/Germany) was used for statistical analyses. ANOVA with post-hoc t-test for independent (PD vs. control) and dependent (t_0_ vs. t_1_) samples was performed, since the variables were widely normally distributed (Kolmogorov-Smirnov-Test). Pearson correlation was used to test for significant correlations. The adjusted level of significance was set as p<0.01.

## Results

**Figure 3:**
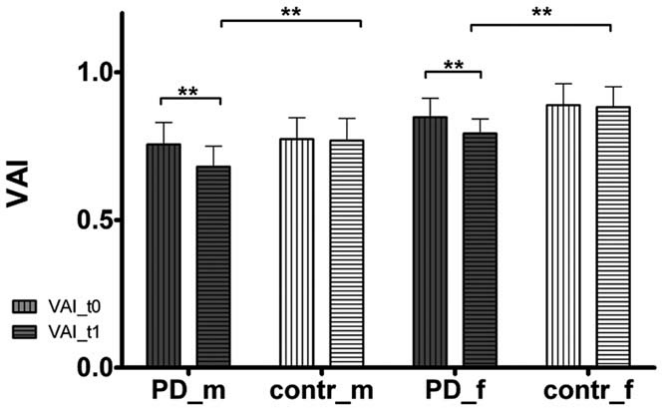
Comparison of vowel articulation index (VAI) at first (VAI_t0) and second (VAI_t1) examination. PD = Parkinson's disease; contr = control group; m = male; f = female. ** = p<0.01.

**Figure 4:**
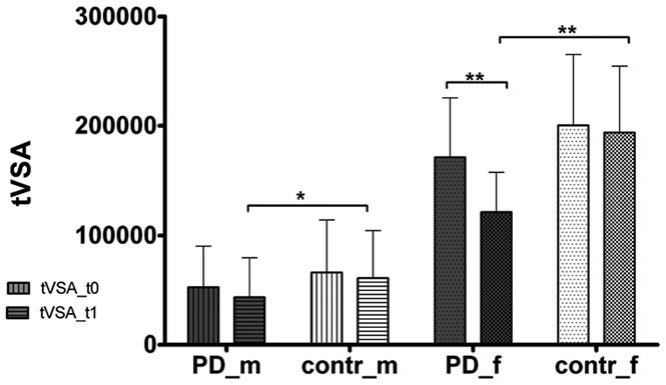
Comparison of triangular vowel space area (tVSA) at first (tVSA_t0) and second (tVSA_t1) examination. PD = Parkinson's disease; contr = control group; m = male; f = female. * = p<0.05; ** = p<0.01.

Detailed numerical data of participants' characteristics and speech parameters are listed in tables 1+2 and 3+4/figure 3 and 4.

**Table 3:** Male participants' characteristics and results.

	PD male (n = 42)					Control male (n = 20)				
	t0		t1		comparison t0 vs. t1	t0		t1		comparison t0 vs. t1
	mean	S.D.	mean	S.D.		mean	S.D.	mean	S.D.	
age	66.27	7.95				68.24	5.09			
t_0_–t_1_ *(months)*			35.02	21.27				21.72	6.25	
disease duration *(months)*	73.57	50.44	108.60	55.87						
levodopa equivalent dosage *(mg)*	561	176	751	226	p<0.0001					
Hoehn&Yahr	1.99	0.30	2.57	0.67	p<0.0001					
UPDRS III	19.48	10.74	19.10	8.28	n.s.					
UPDRS speech item	0.96	0.66	1.36	0.87	p<0.05					
UPDRS axial score	1.50	1.50	2.83	1.75	p<0.0001					
**VAI**	0.756	0.074	0.680	0.070	p<0.0001	0.774	0.072	0.769	0.075	n.s.
**tVSA**	52,700	37,495	43,571	36,009	n.s.	66,053	48,151	60,983	43,432	n.s.

n.s. = not significant; S.D. = standard deviation; Hz = hertz.

UPDRS III = Unified Parkinson's Disease Rating Scale/Motor Score.

VAI = vowel articulation index; tVSA = triangular vowel space area.

**Table 4:** Female participants' characteristics and results.

	PD female (n = 25)					Control female (n = 20)				
	t0		t1		comparison t0 vs. t1	t0		t1		comparison t0 vs. t1
	mean	S.D.	mean	S.D.		mean	S.D.	mean	S.D.	
age	65.48	9.26				67.44	7.60			
t_0_–t_1_ *(months)*			32.44	19.00				21.01	5.99	
disease duration *(months)*	72.96	61.67	105.40	57.35						
levodopa equivalent dosage *(mg)*	566	152	677	166	p<0.0001					
Hoehn&Yahr	2.20	0.41	2.56	0.58	p<0.001					
UPDRS III	18.20	11.03	19.20	8.57	n.s.					
UPDRS speech item	0.76	0.60	1.20	0.71	p<0.05					
UPDRS axial score	1.56	1.26	2.68	1.53	p<0.0001					
**VAI**	0.848	0.064	0.793	0.049	p = 0.001	0.889	0.072	0.882	0.069	n.s.
**tVSA**	171,177	54,258	121,396	35,942	p = 0.0003	200,344	64,888	193,763	60,750	n.s.

n.s. = not significant; S.D. = standard deviation; Hz = hertz.

UPDRS III = Unified Parkinson's Disease Rating Scale/Motor Score.

VAI = vowel articulation index; tVSA = triangular vowel space area.

According to ANOVA, condition (PD vs. control) and gender were shown to be independent factors for tVSA and VAI (p<0.001 respectively). Therefore, a gender-related analysis and comparison with the accordant control group was performed.

### PD group as a whole

No correlations were seen between the LEDs at first and second examination and tVSA and VAI. However, there was a correlation between the difference of the total UPDRS score (ΔUPDRS) and the difference of LED (ΔLED) between t_0_ and t_1_ (R = 0.455, p<0.0001). Concerning H&Y stages and the UPDRS gait subscore, there were only trends to an inverse correlation to ΔLED.

### Male group

No significant differences concerning age at t_0_ were seen between the male PD group and the control group, however, the time interval between first and second examination was shorter in the control group (p<0.001). In the control group, tVSA and VAI remained stable over time, whereas in the PD group, there was a significant reduction of VAI at t_1_ as compared to t_0_ (p<0.0001) which was not observed in tVSA. Inter-group comparison revealed no relevant differences of VAI between PD and control group at t_0_ (p = 0.08), but a significant reduction at t_1_ in the PD group when compared to the control group (p<0.0001). tVSA in the PD group was found to be reduced when compared to the control group only at t_1_ (p = 0.050) without reaching adjusted level of significance.

No correlations were seen between tVSA or VAI and disease duration, time interval between t_0_ and t_1_, UPDRS III and UPDRS speech item. However, there were weak negative correlations between total UPDRS and UPDRS gait subscore and VAI at t_1_ (R = −0.386, p = 0.006 and R = −0.290, p<0.05) and a clear negative correlation between the difference of VAI (ΔVAI) and the difference between the UPDRS gait subscores (ΔUPDRS_gait_) at t_0_ and t_1_ (R = −0.657, p<0.0001). A similar but weaker correlation was seen between ΔUPDRS_gait_ and ΔtVSA (R = −0.273, p = 0.05).

### Female group

No significant differences concerning age at t_0_ were seen between the female PD group and the control group, however, the time interval between first and second examination was shorter in the control group (p = 0.005). In the control group, VAI and tVSA remained stable over time, whereas in the female PD group, there was a significant reduction of VAI at t_1_ as compared to t_0_ (p = 0.001) which was also seen for tVSA (p = 0.0003). Inter-group comparison revealed no significant differences of tVSA and VAI between PD and control group at t_0_ (VAI: p = 0.06; tVSA: p = 0.127), but a significant reduction at t_1_ in the PD group when compared to the control group (VAI: p<0.0001, tVSA: p = 0.0002).

No correlations were seen between tVSA or VAI and disease duration, time interval between t_0_ and t_1_ and UPDRS III, however, global speech impairment according the UPDRS speech item showed a correlation to VAI (but not to tVSA) at t_0_ (R = −0.535, p = 0.003) and t_1_ (R = −0.558, p = 0.002) as well. The UPDRS gait subscore was correlated to VAI at t_0_ (R = −0.508, p = 0.005), but not at t_1_. A similar, but weaker correlation was also found between tVSA_t0_ and the UPDRS gait subscores (R = −0.415, p = 0.019). Furthermore, there was a negative correlation between ΔVAI and ΔUPDRS_gait_ (R = −0.491, p = 0.006) and between ΔtVSA and ΔUPDRS_gait_ respectively (R = −0.537, p = 0.003).

The average formant frequency values for the single vowels are listed in tables 1+2 with a supplementary display of normative data from literature based upon German vowels extracted from speech material of a sample of n = 69 male and n = 58 female subjects [33]. No formal statistical analyses for data comparison were performed in order to avoid the problem of multiple testing. However, there are obvious differences between F1 and F2 values between published data and the present control group with higher F1 values for /α/ and /i/ and lower F2 values for /u/ as the main discrepancy. Calculation of VAI and tVSA values from the published data led to comparable results as in the present study, however, there was an inverse behaviour of VAI and tVSA between male and female speakers with lower VAI and tVSA values in the published female group.

## Discussion

This study analysed the development of vowel articulation as one distinctive parameter of speech in the clinical course of PD. While general motor performance according to UPDRS III remained relatively stable over time (obviously due to an interim adaptation of the dopaminergic medication illustrated by an increase of LEDs between first and second examination), vowel articulation in Parkinsonian speakers exhibited a significant deterioration which was not observed in the control group and therefore can be interpreted as a symptom of disease progression rather than as an effect of aging although – admittedly – the average follow-up interval was shorter in the control than in the PD group. Notwithstanding the widely stable overall motor performance, the majority of patients featured a decline of gait function and an increase of H&Y staging between first and second examination which showed a correlation to the deterioration of vowel articulation. Therefore, one might argue that the progressive impairment of vowel articulation parallels the progression of axial motor symptoms which are not sufficiently improved by the augmentation of dopaminergic medication in the course of the disease.

The vowels for analysis of tVSA and VAI had derived from different standardized words of a reading task to minimize the impact of lexical factors as word frequency and phonological neighborhood density which have been shown to influence vowel space area [34]. However, since the vowels were extracted from different phonemes with diverse impact e.g. of coarticulation phenomena, the calculated average formant frequency values presumably mirror a composition of slightly diverse vowels rather than the “pure” corner vowels, though, with identical effects in the PD and the control group. This methodological aspect might be the explanation for the differences found between the control group of the present study and published data from literature which additionally vary concerning speakers age (which ranged from 20 to 30 years in the cited study) [33].

The reading task was chosen in order to obtain comparable data for the acoustical analysis, although it is well known that several speech modalities as well as overall speech intelligibility are influenced by the underlying speech task [35], [36]. Measurement of VAI had been previously shown to mirror the reduction of “working space for vowels” as a consequence of articulatory undershooting in Parkinsonian speakers even before the manifestation of severe speech impairment [3]. However, in the present study, VAI at t_0_ showed only a tendency to reduction in male and female PD patients which featured only mild overall speech impairment, comparable to the previous investigation of our group [3]. Therefore, the potential of VAI to serve as a very early marker of subclinical dysarthria in PD has to be put into perspective and necessitates further validation. Interestingly, an inverse correlation between VAI and global speech impairment according to UPDRS speech item was seen only in the female PD subgroup which suggests a differential contribution of speech aspects on overall intelligibility among the genders.

These gender-related differences might be due to the sexual dimorphism of the laryngo-pharyngeal tractus with different size and configuration of the tongue, the three dimensional shape and acoustical properties of vocal cord and the resonatory cavities in male and female. According to previous studies in healthy speakers, gender-related differences of overall speech intelligibility had been attributed to these anatomical factors since fundamental and formant frequencies as well as the resulting working space for vowels have been found to vary significantly between healthy men and women [37]–[41]. On the other hand, additional to pure anatomical conditions, gender-differences have been previously documented concerning disturbed prosody in Parkinsonian speakers with a relatively stronger reduction of pitch variability, decreased pause ratio and a tendency to accelerated speech rate in female PD patients only [41]. This finding could serve as a first evidence for diverse profiles of dysarthria in male and female PD speakers which requires further investigation.

One further aim of the current study was the comparison of VAI and tVSA in the monitoring of vowel articulation over time. Interestingly, the aforementioned gender-related differences were also mirrored by the behaviour of tVSA which in female Parkinsonian speakers showed accordant changes as VAI. Besides, in female PD patients, VAI and tVSA showed similar correlations to the UPDRS gait subscore, but only VAI was correlated to the UPDRS speech item. On the other hand, in the male group, tVSA featured no significant differences in the course of the disease and in comparison to male healthy subjects and showed no correlation to the UPDRS gait subscore. Therefore, VAI seems to be superior to tVSA especially in male speakers in the earlier stage of PD, whereas measurement of tVSA and VAI rather seem to be equally applicable in female speakers, in the later stages and for intra-individual comparison. However, this preliminary interpretation of a possible complementary value of tVSA and VAI needs further validation.

Since in the current investigation, all Parkinsonian speakers were under different therapeutic regimen, the results allow no conclusion about a possible effect of dopaminergic stimulation on vowel articulation, although no correlations were found between vowel articulation and the LEDs. While augmentation of LEDs between first and second examination was obviously able to stabilize and sometimes even ameliorate global motor function in the PD group, there was an increase of the UPDRS gait subscore which showed a correlation to the deterioration of vowel articulation in the PD group. In a similar vein, a previous longitudinal investigation of our group revealed an analogue pattern of progression of dysprosody in Parkinsonian speakers – again with some gender-dependent characteristics - independent from global motor impairment [28], but in that study, no subscores of the UPDRS were given. Summarized, these findings give reason to the hypothesis that impairment of vowel articulation and progressive prosodic changes could be the result of an escalation of axial dysfunction too subtle to be mirrored by global UPDRS motor score. Alternatively, alterations of speech parameters could be completely independent from motor performance maybe based upon non-dopaminergic mechanisms, as it is supported by the lack of an unequivocal evidence of speech amelioration under levodopa admission [42]–[44].

One limitation of the present study is that disease duration on first examination as well as period of time between the two examinations were not standardized but lay within a wide range; therefore, it is not appraisable if progression of vowel articulation impairment follows the tempo of motor deterioration. Since mean disease duration on first examination was about 6 years, the current findings seem to locate the phase of articulatory deterioration into a more advanced stage of disease, paralleling the increase of axial symptoms and gait dysfunction. These estimations find some substantiation by a previous study on speech impairment in a large sample of patients with PD in different stages of the disease: While abnormalities of voice were already present in patients with only mild overall motor impairment, additional deterioration of articulation and fluency appeared in the more advanced stages of the disease [23]. On the other hand, subtle telemetric analyses of different speech variables have been successfully used to predict the severity of PD in a pilot study on a large number of 82 patients [45]. However, according to the present data, worsening of speech performance seem to follow an individual pace without clear correlation to progression of motor performance or disease duration, since there were no correlations between changes of tVSA or VAI and the time period passed between the visits.

Summarized, the current study together with the afore mentioned findings justify the assumption that acoustic analyses of vowel articulation and dysprosody could turn out to become a useful instrument for the monitoring of non-dopaminergic disease progression at least in the more advanced stages of PD, since impairment of vowel articulation was found to parallel the increasing deterioration of gait. Additional investigations are needed to clarify and further substantiate a possible differential value of tVSA and VAI measurement in the different gender and different stages of disease. Further longitudinal studies with regard to several distinct speech parameters are warranted with standardized follow-up examinations to obtain further insight into pathophysiology and progression of speech impairment in Parkinson's disease.

## Supporting Information

Supporting Information S1 **Reading passage with labelling of the vowels in bold type which have been extracted for formant frequency measurement.**
(DOC)
