# Supplementary material for: Impairment of Vowel Articulation as a Possible Marker of Disease Progression in Parkinson's Disease
Source: PLoS One. 2012 Feb 28;7(2):e32132. doi: 10.1371/journal.pone.0032132 (PMC3289640; doi:10.1371/journal.pone.0032132)
Supplement: Supporting Information S1 — Reading passage with labelling of the vowels in bold type which have been extracted for formant frequency measurement. (DOC) [file pone.0032132.s001.doc]

„Von einer gew**a**lt**i**gen, von den Behörden geschützten Inv**a**sion wird z**u**r Zeit d**i**e Golf- und Paz**i**fikküste Mexikos heimges**u**cht: Wie **a**lljährlich im J**u**n**i** kommen H**u**nderttausende von Sch**i**ldkröten aus dem Meer, um an L**a**nd ihre Eier abz**u**legen, **a**llein in der Nähe von Tampico w**u**rden etw**a** 5000 Schildkröten beobachtet. Insgesamt wird in den kommenden Wochen mit einer Invasion von mehr als einer halben Million Schildkröten gerechnet. D**i**e mexik**a**n**i**schen Behörden l**a**ssen d**i**e Legeplätze sorgfältig bew**a**chen, **u**m den D**i**ebstahl von Eiern z**u** verh**i**ndern **u**nd ausreichend Schildkrötenn**a**chw**u**chs sicherzustellen.“
